# A variance component method for integrated pathway analysis of gene expression data

**DOI:** 10.1186/s12919-016-0053-6

**Published:** 2016-10-18

**Authors:** Ellen E. Quillen, John Blangero, Laura Almasy

**Affiliations:** 1 Department of Genetics, Texas Biomedical Research Institute, PO Box 760549, San Antonio, TX 78245 USA; 2 South Texas Diabetes and Obesity Center, University of Texas Health Science Center at San Antonio, 7703 Floyd Curl Drive, San Antonio, TX 78229 USA

## Abstract

**Background:**

The application of pathway and gene-set based analyses to high-throughput data is increasingly common and represents an effort to understand underlying biology where single-gene or single-marker analyses have failed. Many such analyses rely on the a priori identification of genes associated with the trait of interest. In contrast, this variance-component–based approach creates a similarity matrix of individuals based on the expression of genes in each pathway.

**Methods:**

We compared 16 methods of calculating similarity for positive control matrices based on probes for the genes used to model the simulated Genetic Analysis Workshop phenotypes.

**Results:**

A simple correlation matrix outperforms the other methods by identifying pathways associated with the simulated phenotypes at nearly twice the rate expected based on the associations of the component transcripts and an approximate false-positive rate of 0.05.

**Conclusions:**

This method has a number of additional advantages compared to single-transcript and pathway overrepresentation analyses, including the ability to estimate the proportion of variation explained by each pathway and the logistical advantage of only calculating the distance matrices once for each messenger RNA data set regardless of the number of phenotypes. Additionally, it offers a significant reduction in the multiple testing burden over individual consideration of each probe.

## Background

Pathway and gene-set enrichment analyses were developed with several goals, including increasing the biological interpretability of genetic association and RNA expression analyses [1]. Because these pathway tests are based on the results of gene- or probe-based prior analyses, they rely on aggregation of individual effects. Here, we developed a method to evaluate the influence of variation in transcript expression data across the pathway as a whole. This has the advantage of implicitly aggregating across effects of individual probes in the pathway, thereby allowing the pathway to become the level of analysis instead of the gene. Additionally, calculating similarity matrices at the pathway level reduces the computational and statistical burden of running association analyses of each probe against each phenotype. To do this, we apply a variance component-based approach to assess the proportion of phenotypic variability explained by similarity matrices constructed from transcript expression data for each gene in a given pathway. Ideally, this method will enable the detection of pathways of significant integrated effect, even if individual transcript levels do not contribute significantly to the phenotypic variation.

## Methods

### Probe association and scaling

In the provided Genetic Analysis Workshop 19 data [2], high-quality transcript abundance data from 20,634 probes generated using the Illumina Sentrix Human Whole Genome (WG-6) arrays was provided for 645 individuals in 20 extended families [3]. Two monozygotic twins were removed from the analysis. Transcript abundance values had been shifted to make the minimum value 1.0 and log_2_ transformed followed by a quantile normalization; we further adjusted the transcript abundance values for sex, age, age^2, and sex*age interaction. The residual values were used for all analyses. Probes were annotated based on their RefSeq IDs and we selected a single, representative probe per gene to avoid upweighting the apparent effect of a gene in the pathway matrix from the inclusion of multiple probes representing a single gene. More than 90 % of genes present in 1 or more pathways are represented by only a single probe. Where there were more than 2 probes mapped to a gene, we compared the expression of each pair of probes using Pearson’s correlation and the probe with the highest average correlation value was considered most representative of the gene as a whole. Where only 2 probes were mapped to a gene, we selected the probe with greater variance. Selected probes were scaled to range between 0 and 1 so that all probes are weighted equally in the similarity calculation; however, weights could be applied at this step to test specific hypotheses or reflect known biological features of the pathway.

### Positive control pathways

Diastolic blood pressure (DBP) values were simulated based in part on genetic variation in *cis*-regulatory and coding variants with a real effect on the messenger RNA (mRNA) probes drawn from the data set [2]. Throughout, only the simulated DBP values from visit 1 in the longitudinal data was considered. Although the mRNA expression levels incompletely explain the phenotypic variation and the simulated phenotypes were modeled from the genetic rather than expression data, the relationships between the genetic and transcript values and between the genetic and phenotype values remains the same among the simulated phenotypes such that there should be consistency in the relationship between transcripts and the phenotype across the 200 simulations of DBP. For all simulations of DBP, the heritability is 0.33. Using SOLAR (Sequential Oligogenic Linkage Analysis Routines) [4], we measured the association of each of the 277 probes representing genes included in the DBP simulation model with the 200 simulated DBP values. We ranked the probes by the number of significant associations (at α = 0.05) across the simulated DBP and created a positive control “pathway” based on each decile.

We generated N × N similarity matrices for the 643 individuals from the probe values in the positive control pathways using 16 methods in the R library *proxy* [5]: correlation [6], cosine similarity (angular) [6], extended Jaccard similarity [7], Bhjattacharyya distance [8], Bray/Curtis dissimilarity [6], Canberra distance [9], Chord distance [10], divergence distance [9], euclidean distance [9], geodesic distance [10], Hellinger distance [11], Mahalanobis distance [12], Manhattan distance [9], Soergel distance [9], Tschebyscheff/Chebyshev distance [9], and Whittaker distance [13]. Where distance metrics were calculated, distances are converted to similarities using the formula 1/(1 + distance).

For each of the 200 replicates, a polygenic null model was generated for simulated DBP and negative-control phenotype Q1 using SOLAR. The Q1 trait, which has a heritability of zero, was modeled independently of transcript and genetic data and should not be associated with any pathway. These polygenic null models include the expected kinship matrix derived from the pedigree with sex and age as covariates and serves as the model to which the similarity matrices are compared. We considered each similarity matrix separately as an additional variance component and applied a likelihood ratio test (LRT) to determine if the positive control pathway explains significantly more of the variation in the phenotype than kinship alone (the null model). For consistency with the individual probe analysis, significance was determined at α = 0.05 for the *p* values derived from the *LRT*. In these analyses, we used the SOLAR-generated 2φ matrix based on expected kinship from the pedigree; however, an empirical kinship matrix generated from other genetic data or a similarity matrix from the full set of probes can be used in place of or in addition to the 2φ matrix [14].

### Pathway selection

For the 5 similarity methods showing the largest number of associations in the positive control pathways across the 200 replicates, similarity matrices were calculated for 723 pathways taken from Pathway Studio 8.0 (Ariadne Genomics Inc., Rockville, MD, USA). The phenotypic variation explained by these similarity matrices in the simulated DBP, Q1, and real DBP values was assessed. These 723 pathways represent a wide variety of basic cellular functions, disease-specific gene sets, immune response, and signaling pathways. However, this method is not limited by the choice of pathway or gene set. It can be applied to any set of probes of interest to the researcher.

## Results

### Comparison of distance calculations

Using a nominal significance threshold (α = 0.05), only 2 genes—*F2RL3* and *B3GAT1*—are independently associated with DBP in more than half of the 200 simulations. The probes in the top decile are associated with DBP in an average of 59.1 simulations (29.6 %), whereas those in the bottom decile have an average of just 2.04 associations in the 200 simulations (1.0 %). The average number of associations falls off steeply beyond the first decile (Table 1).

**Table 1:** Percentage of simulated phenotypes associated with positive control matrices by similarity calculation method

		Simulated DBP					Q1				
Matrix	Average (%)	bhja (%)	corr (%)	dive (%)	ejac (%)	eucl (%)	bhja (%)	corr (%)	dive (%)	ejac (%)	eucl (%)
1	29.6	34.2	65.0	34.0	61.5	36.0	1.0	5.5	3.5	3.5	1.5
2	18.1	23.5	55.0	14.0	50.0	25.0	0.0	3.5	1.5	2.5	0.0
3	13.3	13.2	34.5	18.5	31.5	15.0	1.5	6.5	1.0	5.5	2.0
4	10.6	4.0	35.0	4.5	28.5	8.5	0.5	4.5	0.0	3.5	1.0
5	8.1	4.5	16.0	4.5	14.5	4.5	0.5	4.5	1.5	5.0	0.5
6	6.0	1.0	7.0	3.0	6.0	1.0	1.0	7.0	0.5	4.5	0.5
7	5.2	1.5	7.0	4.5	6.0	2.0	0.5	3.0	1.5	3.0	0.0
8	3.4	0.0	2.5	2.5	2.5	0.5	0.5	3.5	0.0	2.5	0.5
9	2.5	0.0	2.5	2.0	2.5	0.0	1.5	4.0	1.5	4.5	1.5
10	1.0	0.0	0.5	1.0	1.0	0.0	1.0	3.5	1.5	3.0	1.0

*bhja* Bhjattacharyya distance, *corr* correlation, *dive* divergence distance, *ejac* extended Jaccard distance, *eucl* euclidean distance

Table 1 shows the proportion of simulations in which the positive control matrices are associated with DBP. The 5 listed similarity methods—Bhjattacharyya distance (bhja), correlation (corr), extended Jaccard distance (ejac), euclidean distance (eucl), and divergence distance (dive)—outperformed the average number of associations for the probes included in the pathway. As expected, all methods showed more associations with simulated DBP in the higher decile pathways where individual probes were more likely to be associated with DBP. The correlation and extended Jaccard methods substantially outperform the other methods with the highest percentage of associations across the simulations of DBP.

In contrast, there is no pattern of associations of the simulated pathways with the negative control Q1 phenotype. Using an α = 0.05 threshold, the number of false-positive associations is approximately what would be expected. The correlation method appears to have a slightly inflated false-positive rate, although this may be the result of kinship detected by the similarities of expression data in the families that is incompletely accounted for by the expected kinship matrix included as the null model.

### Experimental pathway matrices

**Fig. 1:**
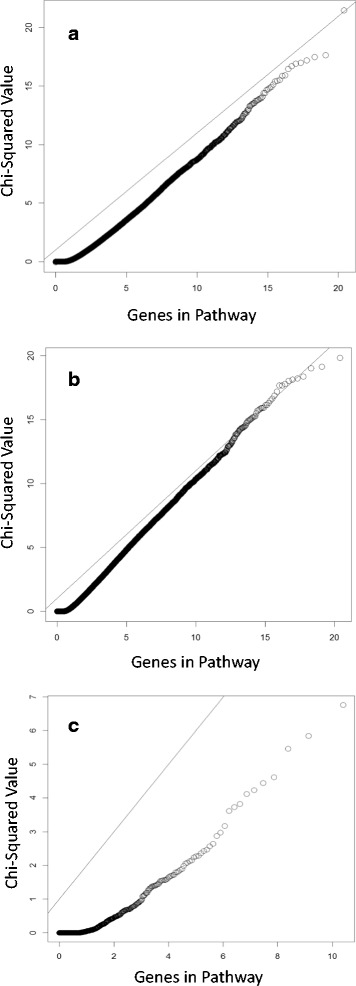
Expected vs. observed *χ*2 values for correlation matrices associated with (**a**) Q1, (**b**) simulated DBP, and (**c**) real DBP

Table 2 shows the formulas for the 5 top similarity methods selected to analyze the Pathway Studio pathways. None of the Ariadne pathway matrices were significantly associated with DBP in more simulations than the top 3 positive control pathways. This is to be expected as the experimental pathways are unlikely to contain exclusively relevant genes. However, several real pathways calculated using the extended Jaccard and basic correlation methods were significant at frequencies similar to that seen for the fourth or fifth decile pathway (20 to 30 % or simulations). These methods also performed best for the control pathways. With the exception of the divergence method, which shows deflation, results from the Pathway Studio pathways show inflation (λ = 1.20 to 1.44) when associated with the DBP simulations but not with Q1. Figure 1 shows the observed versus expected *χ* ^2^ values for the correlation method for simulated Q1 (λ = 1.01) and DBP (λ = 1.25), as well as the real data. The correlation method produces the best results with 5 Pathway Studio matrices significantly associated with DBP in 20 to 25 % of simulations. The top Pathway Studio pathways are reasonable candidates with 6.7 % of genes in the pathways included as causal probes in the model. One pathway calculated with the correlation method is significantly associated with simulated DBP, despite containing no probes independently associated with the simulated phenotypes. Although this appears to be a false positive, it is possible that the genetic variants underlying these probes are in linkage disequilibrium with 1 or more genetic variants that formed the basis of the simulation.

**Table 2:** Formulas for selected distance matrix calculations

Method	Formula
Bhjattacharyya distance	sqrt(sum_i_ (sqrt(x_i_)–sqrt(y_i_))^2^)
Correlation	xy/sqrt(xx * yy) for centered x,y
Divergence distance	sum_i_ (x_i_-y_i_)^2/(x_i_ + y_i_)^2^
Euclidean distance	sqrt(sum_i_ (x_i_-y_i_)^2^))
Extended Jaccard distance	xy/(xx + yy-xy)

### Real diastolic blood pressure

When the 5 similarity methods are applied to the real DBP data, the minimum *p* values for the LRT are approximately 5 × 10^−3^, failing to surpass the Bonferroni-corrected threshold for 723 pathways. This may be a result of the relatively low heritability of DBP (0.33). Twelve pathways show a nominally significant result: Focal junction assembly, cleavage of lamina in apoptosis, systemic lupus erythematosus, glycan catabolism, TGFi, OA transport, fatty acid biosynthesis, NF-1, Myc Mad Max, type 1 diabetes mellitus, and triacylglycerols degradation. The first 3 of these pathways contain probes with previous associations with DBP in this data set. Any analysis of gene expression data must consider the directionality of effect. The appearance of pathways involved in the production and regulation of glycans, triacylglycerols, and fatty acids may be from a phenotypic correlation between individuals with high blood pressure and cardiovascular disease or other outcomes related to the metabolic syndrome. Regardless of the directionality of effect, their appearance among the top pathways is sensible.

The proportion of phenotypic variance explained by each pathway can also be obtained from comparing the null model to one including the matrix-derived variance component. The majority of the nominally significant pathways explain approximately 1 % of the overall variation in DBP. However, 2 large pathways—focal junction assembly and systemic lupus erythematosus—explain 4 and 6 % of the phenotypic variation, respectively. Each of these pathways contains 2 probes independently associated with DBP.

**Fig. 2:**
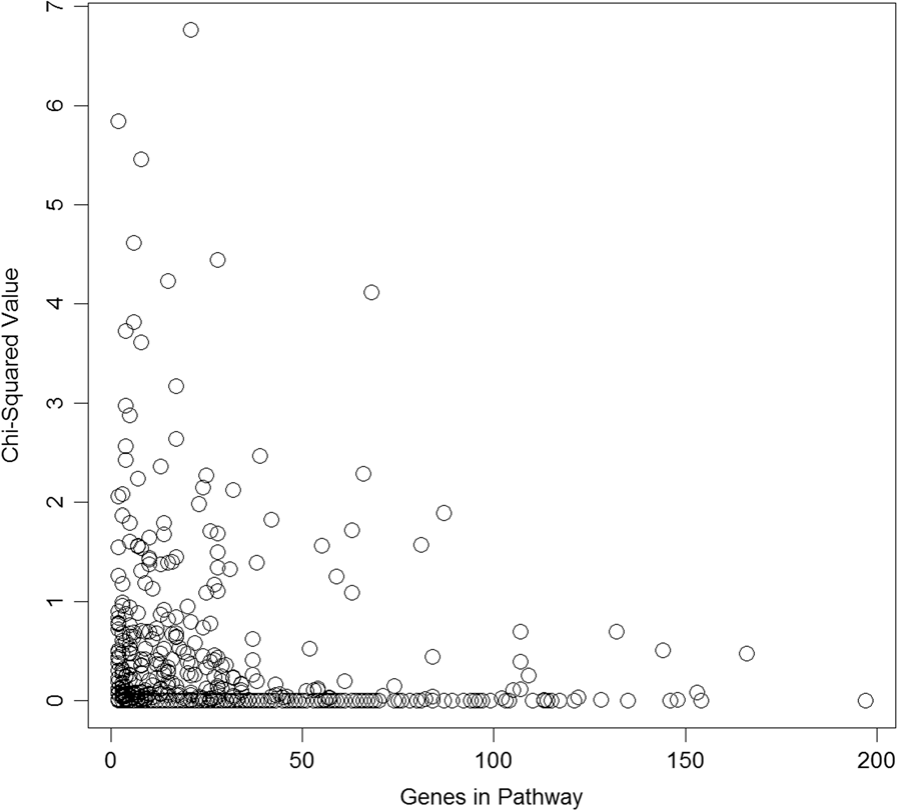
Pathway size vs. chi-squared value in real data

To determine if larger pathways were simply capturing more of the total transcript variation and were therefore more likely to be associated with any phenotype, the *χ* ^2^ values for the real DBP were plotted against pathway size (Fig. 2). No correlation was seen.

## Discussion

The relatively weak relationship between the simulated DBP values and the transcript data makes this a more conservative test than if DBP had been modeled directly from the expression data. Despite this limitation, the use of similarity matrices generated from sets of probes in a variance component-based pathway approach outperformed a single-probe association test. Specifically, the observed number of associations with simulated DBP was higher for the correlation and extended Jaccard similarity matrices for positive control pathways containing probes for genes modeled to be causative than the average for the probes contained in those same control pathways.

Nominal *p* values were used across all analyses for similarity of comparison between individual probe associations and pathway similarity matrices, but consideration of the *p* values illustrates the reduction in the number of tests when the pathway becomes the unit of initial analysis. In the single-probe analyses, none of the 17,265 probes reached a Bonferroni-corrected *p* value of 2.9 × 10^−6^. In contrast, multiple pathways surpassed the Bonferroni-corrected critical *p* value of 6.9 × 10^−5^ for the simulated phenotypes across all similarity methods. When the correlation method is used to assess the effect of the Pathway Studio pathways on the real DBP phenotype, none of the pathways are significantly associated after multiple testing. However, several of the nominally significant pathways are plausible candidates for contributing to DBP. In addition to significantly reducing the multiple testing burden, this method, like all pathway-based tests, also serves to identify potentially important biological pathways instead of isolated genes.

Although this method should allow for the detection of pathways containing a large number of genes just below the significance threshold, it is difficult to clearly differentiate these associations from false positives based on this simulation. Additionally, the method of equally weighting the probes is problematic with pathways containing large numbers of genes as it may dilute the effect of these genes of moderate effect. Weighting based on additional biological information could improve the performance. Furthermore, the method will likely be more effective where the heritability of the phenotype is higher and expression explains a larger proportion of the variance.

## Conclusions

The use of a correlation matrix to generate variance components for pathways or gene-sets provides a means for detecting multiple genes that together contribute to phenotypic variation but cannot be detected individually. The correlation matrix is simple to calculate from any type of input and the same matrices can be used to analyze all available phenotypes in a data set, saving computation time. Additionally, the LRT is straightforward to implement in SOLAR with a single additional variance component, but more complex models incorporating multiple pathways or using an empirical kinship matrix as a null model could be incorporated. As data sets grow, this method, applied to transcript or genotypic data, provides a useful method for prioritizing biological pathways for further investigation while avoiding the multiple-testing burden.
